# Control of fibrosis and hypertrophic scar formation via glycolysis regulation with IR780

**DOI:** 10.1093/burnst/tkac015

**Published:** 2022-06-24

**Authors:** Xinxian Meng, Zhixi Yu, Wanyu Xu, Jun Chai, Shuo Fang, Peiru Min, Yunsheng Chen, Yixin Zhang, Zheng Zhang

**Affiliations:** Department of Plastic and Reconstructive Surgery, Shanghai Ninth People's Hospital, School of Medicine, Shanghai Jiao Tong University, 639 Zhizaoju Rd, Shanghai 200011, China; Department of Plastic and Reconstructive Surgery, Shanghai Ninth People's Hospital, School of Medicine, Shanghai Jiao Tong University, 639 Zhizaoju Rd, Shanghai 200011, China; Department of Plastic and Reconstructive Surgery, Shanghai Ninth People's Hospital, School of Medicine, Shanghai Jiao Tong University, 639 Zhizaoju Rd, Shanghai 200011, China; Department of Plastic and Reconstructive Surgery, Shanghai Ninth People's Hospital, School of Medicine, Shanghai Jiao Tong University, 639 Zhizaoju Rd, Shanghai 200011, China; Department of Plastic and Reconstructive Surgery, The Affiliated Suzhou Hospital of Nanjing Medical University, Suzhou Municipal hospital, Gusu school, Nanjing Medical University, Suzhou 215008, Jiangsu, China; Department of Plastic and Reconstruction, First Affiliated Hospital of Naval Medical University, Shanghai 200433, China; Department of Plastic and Reconstructive Surgery, Shanghai Ninth People's Hospital, School of Medicine, Shanghai Jiao Tong University, 639 Zhizaoju Rd, Shanghai 200011, China; Department of Plastic and Reconstructive Surgery, Shanghai Ninth People's Hospital, School of Medicine, Shanghai Jiao Tong University, 639 Zhizaoju Rd, Shanghai 200011, China; Department of Burns and Plastic Surgery, Shanghai Institute of Burns Research, Ruijin Hospital Affiliated to Shanghai Jiao Tong University School of Medicine, Shanghai 200025, China; Department of Plastic and Reconstructive Surgery, Shanghai Ninth People's Hospital, School of Medicine, Shanghai Jiao Tong University, 639 Zhizaoju Rd, Shanghai 200011, China; Department of Plastic and Reconstructive Surgery, Shanghai Ninth People's Hospital, School of Medicine, Shanghai Jiao Tong University, 639 Zhizaoju Rd, Shanghai 200011, China

**Keywords:** Hypertrophic scar, Glycolysis, Fibrosis, IR780, Activated fibroblast

## Abstract

**Background:**

Hypertrophic scars (HS) represent one of the most common clinical challenges due to unsatisfactory therapeutic results. HS formation is associated with the abnormal activation of fibroblasts and their excessive fibrotic behavior. Glycolysis dysregulation has been shown to participate in the incidence and progression of various fibrotic diseases and shows potential as a means of controlling HS formation. This work aimed to discuss the impact of augmented glycolysis on HS and to propose a method for controlling HS formation through glycolysis regulation.

**Methods:**

Here, augmented glycolysis was confirmed together with enhanced fibrotic activity in both HS fibroblasts (HFs) and HS tissues, and the suppression of glycolysis also attenuated fibroblast activation. We also introduced IR780, a heptamethine cyanine dye, to regulate glycolysis for the control of HS formation.

**Results:**

*In vitro*, cell studies indicated that IR780 significantly down-regulated glycolysis and suppressed the fibrotic activity of HFs. *In vivo,* the intralesional injection of IR780 into rabbit HS models led to the downregulation of glycolysis and the control of HS formation. Furthermore, IR780 accumulated preferentially in activated fibroblasts in both *in vitro* and *in vivo* studies, and thus specifically downregulated glycolysis and efficiently controlled fibrosis by targeting activated fibroblasts.

**Conclusions:**

This work identified a strategy for controlling fibrosis and HS formation from the perspective of glycolysis regulation with IR780 targeting of activated fibroblasts.

Highlights

- Augmented glycolysis is verified in HS and is required for excessive fibrotic behavior of HS fibroblasts.
- These activated fibroblasts show augmented glycolysis and fibrosis and are responsible for HS formation.
- IR780 can preferentially accumulate in the activated fibroblasts and downregulate glycolysis, thus controlling fibrosis and HS formation.

## Background

Hypertrophic scar (HS) is a type of pathological scar that is characterized by persistent dermal fibrosis and attributed to abnormally activated fibroblasts [1,2]. Unfortunately, due to the complex mechanism of HS formation, it remains a common challenge in clinical settings with unsatisfactory therapeutic results, and control of fibrosis during HS formation is required [3,4]. Although various hypotheses exist to explain the pathogenesis of HS, it is commonly believed that the major contributors are the abnormal activation of fibroblasts and the subsequent synthesis of extracellular matrix **(**ECM). The activation of fibroblasts involves transformation from a relatively quiescent state to an activated and excessively ECM-producing state. This activation is temporary during the normal wound healing process but persists during HS formation [5]. As a result, targeting activated fibroblasts and controlling their fibrotic behavior is the main strategy for controlling HS formation. Recently, energy metabolism has become a particularly interesting topic in fibrotic diseases. Emerging evidence reveals the metabolic shift from mitochondrial oxidative phosphorylation to aerobic glycolysis during the progression of various organic fibrotic diseases, thus indicating the underlying profibrotic effect of metabolic dysregulation during fibroblast activation [6–8]. This metabolic shift, also known as the Warburg effect, acts as an adaptative mechanism to support augmented biosynthetic requirements following fibroblast activation [9–11]. Scientists have also investigated glycolysis in pathological scars, particularly keloid scars (KS). The augmentation of glycolysis has been demonstrated in fibroblasts extracted from KS [12,13], and a higher level of glycolysis can act as an indicator for an increased risk of keloid formation after burns [14]. Collectively, this evidence indicates that the regulation of augmented glycolysis may become a potential solution for controlling fibrosis and tissue scarring during the formation of HS.

Various anti-glycolytic agents, particularly 2-deoxy-d-glucose (2DG, a glucose analog that targets hexokinase as a competitive inhibitor), were proposed and proven to be beneficial in controlling organic fibrotic diseases [10,15,16]. However, their clinical translation is significantly impeded by their non-specificity. They will also act on normal cells and influence their basic functionality. As a result, this lack of specificity can cause systemic toxicity and thus limit the maximal tolerated dose [10]. Furthermore, previous studies suggest that heterogeneity exists within pathological scars and that only a certain proportion of fibroblasts are activated and present with higher levels of glycolysis, thus playing a crucial role in the production of ECM and the formation of scars [17,18]. As a result, anti-glycolytic agents that specifically target these activated and highly glycolytic cells should be more suitable for controlling scar formation. Recent studies showed that IR780, a heptamethine dye, could down-regulate the expression of glycolysis-associated genes [19,20]. More importantly, it was proven that IR780 can selectively accumulate in cells with high fibrotic activity via organic anion transporter peptides (OATPs) [19,21,22]. This preferential accumulation may help to achieve higher levels of precision in terms of regulation and avoid the side-effects caused by non-specificity. Collectively, it is indicated that IR780 may represent a new candidate for controlling HS formation by glycolysis regulation.

**Figure 1.**
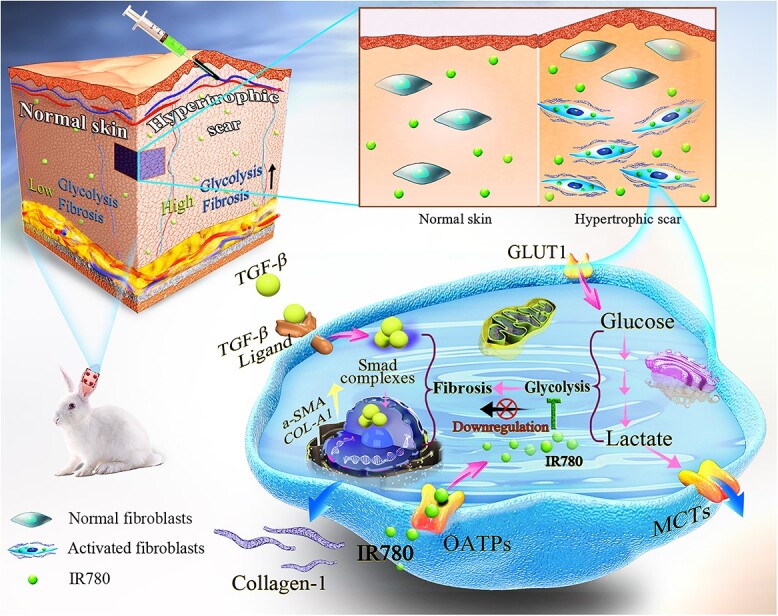
Schematic illustration of controlling fibrosis and hypertrophic scar formation via glycolysis regulation with IR780. *α-SMA* alpha smooth muscle actin, *COL1A1* collagen type I alpha1, *GLUT1* glucose transporter-1, *MCT* monocarboxylate transporter, *OATPs* organic anion transporter peptides, *TGF-β1* transforming growth factor-β1

Although HS are the predominant type among pathological scars following burns and trauma, previous studies have rarely investigated the metabolic dysregulation underlying HS formation. Furthermore, HS are believed to involve different a pathogenic mechanism and have a different clinical appearance when compared with KS, and previous findings for KS should not simply be applied to HS [23]. Consequently, there is a critical need to investigate how HS formation is related to augmented glycolysis and whether glycolysis regulation could affect HS formation. In this study, with IR780 as a potential anti-glycolytic agent targeting activated fibroblasts, we evaluate its glycolysis regulation in HS and investigate how this process controls fibrosis and HS formation both *in vitro* and *in vivo* (Figure 1)*.*

## Methods

### Reagents

IR780 was obtained from Sigma-Aldrich (Darmstadt, Germany), 2DG was obtained from MedChemExpress (MCE, Monmouth Junction, USA), human recombinant transforming growth factor-β1 (anti-TGF-β1) antibody was obtained from Peprotech (Cranbury, USA) and sulfobromophthalein disodium salt hydrate (anti-BSP) was from Sigma-Aldrich. Antibodies of hexokinase-II (anti-HK2), anti-pyruvate kinase isozyme M2 (anti-PKM2), anti-lactate dehydrogenase A (anti-LDHA), anti-glucose transporter-1 (anti-GLUT1), anti-collagen type I alpha1 (anti-COL1A1), anti-fibronectin, anti-alpha-smooth muscle actin (anti-α-SMA) and anti-solute carrier organic anion transporter family member 2A1 (anti-SLCO2A1) antibodies were purchased from Abcam (Cambridge, UK). Anti-glyceraldehyde-3-phosphate dehydrogenase (anti-GAPDH) was obtained from Proteintech (Rosemont, USA). Goat anti-rabbit secondary antibodies were obtained from LI-COR Biosciences (Lincoln, USA).

### Sample collection and cell culture

Samples of normal skin (NS), HS and KS were obtained from patients attending the Department of Plastic and Reconstructive Surgery, Shanghai Ninth People’s Hospital, Shanghai Jiao Tong University (this research was approved by the Medical Ethics Committee). Tissues were collected in sterile bottles and kept at 4°C before processing. The dermis of each specimen was separated and cut into 1 mm^3^ pieces; these were then digested with 2 mg/ml of collagenase type I (Gibco, Thermo-Fisher, Fredrick, USA) at 37°C for 4 h with shaking to achieve a single cell suspension. The extracted fibroblasts [NS fibroblasts (NFs), HS fibroblasts (HFs) and keloid fibroblasts (KFs)] were cultured in Dulbecco’s modified Eagle’s medium (DMEM; Gibco) with 10% fetal bovine serum (Gibco), 100 U/ml penicillin and 100 μg/ml streptomycin (Gibco) at 37°C with 5% CO_2_. Cells from passages 2–4 were used in the subsequent experiments.

### Rabbit HS models

Rabbit HS models were constructed for *in vivo* studies with the approval of the Animal Experimentation Ethics Committee of the School of Medicine, Shanghai Jiao Tong University. Adult New Zealand white rabbits (2.0–2.5 kg; Si-Lai-Ke, Shanghai, China) were anesthetized by the intravenous delivery of pentobarbital sodium (30 mg/kg). Four wounds (10 mm in diameter) were created on each ear by removing the skin and the perichondrium on the ventral side. The rabbits were then raised individually under conditions of controlled temperature and humidity. Before scar excision, the rabbits were euthanized by injecting an overdose of pentobarbital sodium.

### Biocompatibility of IR780

Cells were seeded onto 96-well plates in triplicate at a density of 5 × 10^3^ cells per well and were then treated with IR780 at different concentrations (0, 2.5, 5, 7.5, 10, 12.5, 15 and 20 μM) for 24 h. Cell viability was detected by Cell Counting Kit-8 (Dojindo, Kumamoto, Japan) assays in accordance with the manufacturer’s guidelines. Cell Counting Kit-8 reagent (10 μL) was added to each well and incubated at 37°C for 1.5 h. The absorbance at 450 nm was read on a microplate reader (Infinite 200 PRO series, TECAN, Research Triangle Park, Switzerland).

### Cell treatment

Cells were treated with different factors to investigate their relative effects on fibrosis. (1) 2DG group: cells were treated with glycolysis inhibitor 2DG (5 mM) for 24 h to suppress glycolysis. (2) *HK2* group: small interfering RNA (siRNA) targeting *HK2* was used to transiently knockdown the glycolytic phenotype and was transfected using lipofectamine 3000 (Invitrogen, Thermo-Fisher, Fredrick, USA) in accordance with the manufacturer’s protocol; the siRNA sequences can be found in Supplementary Table S1 (see online supplementary material). (3) IR780 group: cells were treated with IR780 (10 μM) for 24 h. (4) TGF-β1 group: the fibrotic phenotype of cells was induced with TGF-β1 (2 ng/ml). HFs were treated with TGF-β1 for 24 h in the TGF-β1 group. To investigate the inhibition ability of 2DG, siRNA targeting *HK2* and IR780 on TGF-β1-induced fibrosis, HFs were further treated as above following 24 h of TGF-β1 treatment.

### Detection of glucose consumption, lactate production and ATP production

Cells (1 × 10^5^) were seeded onto 6-well plates in triplicate and cultured at 37°C. The culture medium was replaced the next day and collected after incubation for 48 h. Then, the glucose and lactate concentrations in the culture supernatant were determined using a glucose assay kit and a lactic acid assay kit (Jiancheng Bioengineering Institute, Nanjin, China). Cell culture supernatant (10 μL) was mixed with glucose assay reagent or lactic assay reagent, respectively, and incubated at 37°C for 10 min. ATP production was measured by the luciferin–luciferase method with an ATP detection kit (Beyotime Biotechnology, Shanghai, China). Finally, the absorbance was read on a microplate reader (Infinite 200 PRO series).

### *In vitro* cell uptake of IR780

To test the different uptake capacity of NFs, HFs and KFs, the cells were seeded, respectively, onto 12-well plates with cell climbing slices in triplicate at a density of 5 × 10^3^ cells per well. Cells were then incubated with 10 μM IR780 in DMEM for 20 min at 37°C. Following three washes with phosphate buffer solution, the cells were fixed by 4% polyoxymethylene for 15 min and mounted with DAPI (4′,6-diamidino-2-phenylindole) Fluoromount (SouthernBiotech, Birmingham, USA). Near-infrared (NIR) images were then captured by confocal laser scanning microscopy (CLSM; Leica, Germany, λex/λem: 630 nm/730 nm). Further, to test the factors that might affect the uptake of IR780, 5 × 10^3^ HFs were seeded in 12-well plates with cell climbing slices and cells were treated with different factors: (1) glycolysis, cells were treated with 2DG (5 mM) for 24 h or siRNA targeting *HK2*, respectively and (2) OATPs, cells were treated with BSP (250 μM) for 30 min. Cells were then incubated with IR780, mounted and imaged, as described above.

### *In vivo* treatment protocol

Rabbits were randomly divided into three groups: the 2DG group (*n* = 3) and IR780 group received an intralesional injection of 2DG (5 mM) and IR780 (10 μM), respectively; the control group (*n* = 3) received an intralesional injection of normal saline only. Rabbits were treated every three days for 4 weeks after re-epithelialization and morphological changes in the scars were recorded with a digital camera (Canon, Ohta-ku, Japan).

### *In vivo* detection of IR780 uptake

Following intradermal injection with IR780 (10 μM), scars were excised at 2, 6, 12 and 24 h after injection. Excised HS tissue was then subjected to cryostat sectioning (10 μm thickness, perpendicular to the surface), fixation with polylysine-coated glass slides and incubation with DAPI for nuclear staining. The *in vivo* distribution of IR780 was investigated by CLSM (DAPI, λex/λem: 358 nm/461 nm; IR780, λex/λem: 630 nm/730 nm).

### RNA extraction and real-time quantitative PCR

Total RNA was extracted using TRIzol reagent (Invitrogen) in accordance with the manufacturer’s protocol. Then, 1 μg of total RNA was reverse transcribed into cDNA using the FastKing cDNA synthesis kit (Tiangen Biotech, Beijing, China). Quantitative PCR was performed with TB Green Premix (Takara, Shiga, Japan). The mRNA expression levels of key glycolytic enzymes were then detected, including HK2, PKM2, LDHA, GLUT1, COL1A1, fibronectin, α-SMA and SLCO2A1. PCR primers were designed based on sequences from corresponding genes (Table S2, see online supplementary material). All data were normalized to the control using β-actin as the internal control.

### Protein extraction and western blotting

Total proteins from cells or tissues were extracted using RIPA lysis buffer containing phosphatase and protease inhibitors (Beyotime Biotechnology). The concentration of total protein was detected with a BCA Protein Assay kit (Beyotime Biotechnology). Equal amounts (20 μg) of protein were then separated using 4–20% sodium dodecyl sulfate polyacrylamide gel electrophoresis gels. Proteins were then transferred to nitrocellulose membranes (0.45 μm; Merck Millipore, Darmstadt, Germany). The membranes were blocked with 5% non-fat milk for 1 h at room temperature and incubated overnight at 4°C with primary antibodies (1:1000). After washing, the membranes were incubated with goat anti-rabbit secondary antibodies (1:5000). Finally, the membranes were visualized using the Odyssey CLx Infrared Imaging System (LI-COR Biosciences). Levels of the GAPDH protein were used as an internal control.

**Figure 2.**
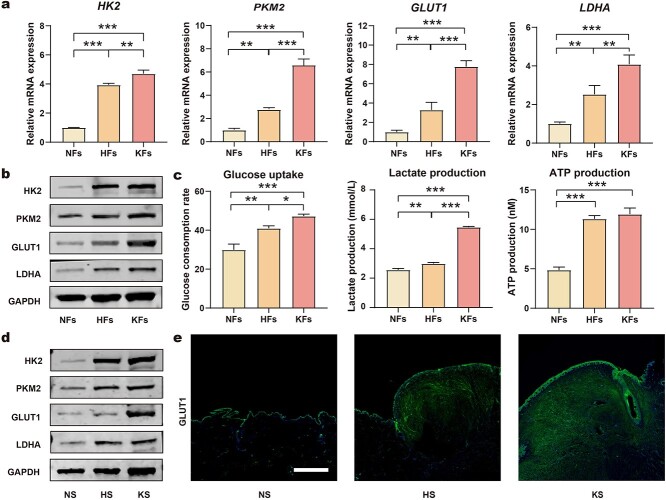
Augmented glycolysis in HFs and HS tissues. (**a**) mRNA levels of HK2, PKM2, GLUT1, and LDHA in NFs, HFs and KFs (*n* = 3). (**b**) Protein levels of HK2, PKM2, GLUT1 and GAPDH in NFs, HFs and KFs. (**c**) Levels of glucose uptake, lactate production and ATP production in NFs, HFs and KFs (*n* = 6). (**d**) Protein levels of HK2, PKM2, GLUT1 and GAPDH in tissue samples of NS, HS and KS. (**e**) Immunofluorescence staining of GLUT1 in tissue samples of NS, HS and KS (scale bar: 2 mm). ^*^p < 0.05, ^**^p < 0.01, ^***^p < 0.001. *HK2* hexokinase-II, *PKM2* pyruvate kinase isozyme M2, *GLUT1* glucose transporter-1, *LDHA* lactate dehydrogenase A, *GAPDH* glyceraldehyde-3-phosphate dehydrogenase, *NFs* normal skin fibroblasts, *HFs* hypertrophic scar fibroblasts, *KFs* keloid fibroblasts, *NS* normal skin, *HS* hypertrophic scar, *KS* keloid scar

### Histological analysis

Paraffin-embedded sections were dehydrated and stained with Masson’s trichrome and Sirius red stain to allow the investigation of collagen deposition. Masson’s and Sirius red solution (Servicebio, Wuhan, China) were applied to sections according to the manufacturer’s instructions. Images were viewed under a microscope and analyzed by Image J. Immunohistochemistry (IHC) and Immunofluorescence (IF) analysis of GLUT1 and α-SMA were also performed. After antigen retrieval and blocking, sections were co-incubated with primary antibodies (1:200) at 4°C overnight, followed by incubation with an appropriate secondary antibody (1:2000) for 1 h. For IHC staining, the sections were counterstained with hematoxylin. For double-labeling IF, the samples were washed and mounted with DAPI Fluoromount (SouthernBiotech) to enable visualization of nuclei, followed by CLSM. Images were acquired and analyzed by Image J.

### Statistical analyses

Primary data are presented as means ± standard deviations and were analyzed in Graphpad Prism 8. Statistical analyses were performed using the unpaired two-tailed Student’s t-test and one-way or two-way analysis of variance. The threshold of statistical significance is set to *p* < 0.05; in all cases, *p* < 0.05 was considered statistically significant and asterisks denote statistical significance (ns, no significance; ^*^p < 0.05; ^**^p < 0.01; ^***^p < 0.001).

**Figure 3.**
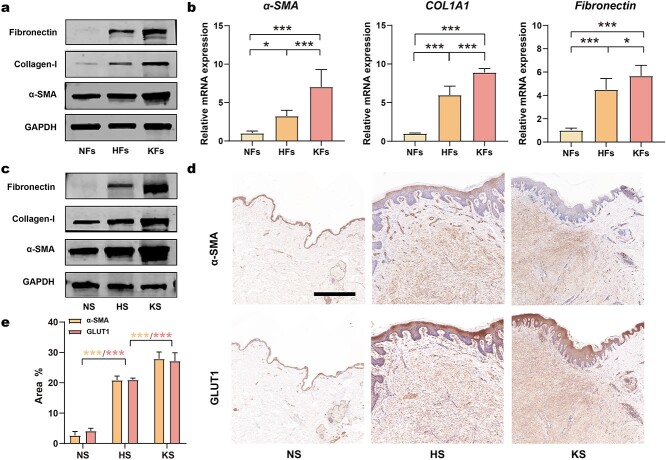
Consistent upregulation of fibrosis and glycolysis in HFs and HS tissue. (**a**) Protein levels of fibronectin, collagen-1, α-SMA and GAPDH in NFs, HFs and KFs. (**b**) mRNA levels of α-SMA, COL1A1 and fibronectin in NFs, HFs and KFs (*n* = 3). (**c**) Protein levels of α-SMA, collagen-1, fibronectin and GAPDH in tissue samples of NS, HS and KS. (**d**, **e**) IHC and quantitation analysis of GLUT1 and α-SMA in tissue samples of NS, HS and KS (*n* = 3; scale bar: 1 mm). ^*^p < 0.05, ^**^p < 0.01, ^***^p < 0.001. *α-SMA* alpha-smooth muscle actin, *COL1A1* collagen type I alpha1, *GAPDH* glyceraldehyde-3-phosphate dehydrogenase, *GLUT1* glucose transporter-1, *NFs* normal skin fibroblasts*, HFs* hypertrophic scar fibroblasts*, KFs* keloid fibroblasts*, NS* normal skin*, HS* hypertrophic scar*, KS* keloid scar, *IHC* immunohistochemistry

## Results

### HS exhibit increased levels of glycolysis

The upregulation of glycolysis has been proven to influence the severity of organ fibrosis and has also been observed in KS. However, few studies have focused on the role of glycolysis during HS formation. Therefore, glycolysis activity was determined in different fibroblasts by measuring the mRNA expression of several glycolysis-related genes (HK2, PKM2, GLUT1 and LDHA). HFs presented significantly increased levels of glycolysis compared with NFs. Interestingly, KFs showed even higher glycolysis than HFs (Figure 2a). These results were supported by the levels of glycolysis-related proteins (Figure 2b; Figure S1a, see online supplementary material). Furthermore, augmented glycolysis was also characterized by the enhanced conversion of glucose into lactate to support higher energy production. As shown in Figure 2c, the levels of glucose consumption, lactate production and ATP production were coordinated with the expression of glycolytic-related genes and proteins.

Furthermore, tissue samples also provided evidence of increased glycolysis in HS. As shown in Figure 2d and Figure S1b, there were markedly increased expression levels of glycolytic-related proteins in both HS and KS in comparison with NS; these results were consistent with those from cellular experiments. To thoroughly characterize the localization of highly glycolytic cells in tissue samples, the expression levels of the facilitative glucose transporter GLUT1 were measured by IF analysis to verify the increased glucose uptake. As shown in Figure 2e, a significant number of fibroblasts showing high expression levels of GLUT1 accumulated within the peripheral regions of both HS and KS, whereas these fibroblasts were rarely found in NS. Furthermore, there were more GLUT1-activated fibroblasts in KS than HS (Figure S2, see online supplementary material).

Collectively, these data demonstrated that glycolysis was reprogrammed to a higher level within HS, but to a milder extent.

### Glycolysis regulates fibrosis during HS formation

Numerous lines of evidence have demonstrated that fibroblasts are activated with augmented fibrotic behavior and are responsible for the large amount of ECM produced during HS formation. Herein, the fibrosis severity and its relationship with glycolysis activity were investigated. Firstly, enhanced fibrosis was detected in both cells and tissue samples by determining the levels of fibrotic markers (α-SMA, fibronectin and collagen-1). All three markers were highly elevated in HFs at both the mRNA and protein levels (Figure 3a, b; Figure S1c). A significant up-regulation of fibrotic markers in HS tissues was also verified by western blotting (Figure 3c; Figure S1d). These results were consistent with the increase in glycolysis activity, thus indicating that there is an underlying synchronous relationship between the expression of fibrotic markers and glycolysis. To further investigate this relationship, the expression of α-SMA and GLUT1 in different tissue samples was studied using IHC staining. Highly consistent immunopositivity for α-SMA and GLUT1 were identified in both HS and KS, whereas neither of these proteins was found in NS (Figure 3d, e). This result was also supported by IF colocalization experiments (Figure S3, see online supplementary material). These findings indicated that most α-SMA-positive fibroblasts in HS were highly glycolytic, thus confirming that fibroblast activation was accompanied by an increase in glycolysis. However, the relationship between enhanced glycolysis and fibrosis remains unknown.

**Figure 4.**
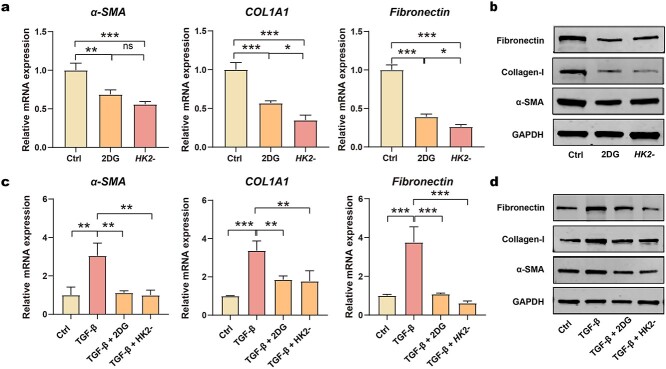
Fibrosis mediated by glycolysis. (**a**, **b**) mRNA levels and protein levels of α-SMA, COL1A1 and fibronectin in HFs treated with 2DG and siRNA targeting *HK2* (*n* = 3); (**c**, **d**) mRNA levels and protein levels of α-SMA, COL1A1 and fibronectin in HFs that were activated with TGF-β1 and then treated with 2DG and siRNA targeting *HK2* (*n* = 3). ^*^p < 0.05, ^**^p < 0.01, ^***^p < 0.001. *Ctrl* control, *2DG* 2-deoxy-d-glucose, *TGF-β1* transforming growth factor-β1, *HK2* small interfering RNA targeting hexokinase-II, *α-SMA* alpha-smooth muscle actin, *COL1A1* collagen type I alpha1, *GAPDH* glyceraldehyde-3-phosphate dehydrogenase

Next, we investigated whether glycolysis was necessary for fibrosis during HS formation (Figure 4). First, we identified the potential functional effect of glycolysis on the activation of fibroblasts. The level of fibrosis was suppressed when cells were treated with 2DG or siRNA targeting *HK2,* thus suggesting that the inhibition of glycolysis could attenuate fibroblast activation (Figure 4a, b; Figure S5a). In addition, the impact of blocked glycolysis on TGF-β1-induced fibroblast activation was also monitored (Figure 4c, d; Figure S5b). As expected, by inhibiting glycolysis, 2DG treatment and the knock-down of *HK2* abolished the TGF-β1-induced increase in fibrosis.

Collectively, these results verified that the activation of fibroblasts in HS was closely associated with the enhanced glycolysis, and the inhibition of glycolysis can suppress the activation of fibroblasts.

### Regulation of fibrosis in fibroblasts via IR780-regulated glycolysis

**Figure 5.**
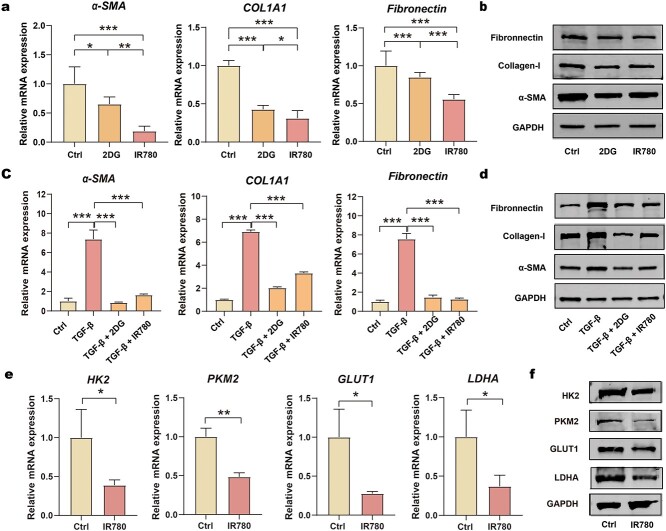
Control of fibrosis via glycolysis regulation with IR780. (**a**, **b**) mRNA levels and protein levels of α-SMA, COL1A1 and fibronectin in HFs treated with 2DG and IR780 (*n* = 3); (**c**, **d**) mRNA levels and protein levels of α-SMA, COL1A1 and fibronectin in HFs are activated with TGF-β1 and then treated with 2DG and IR780 (*n* = 3); (**e**, **f**) mRNA levels and protein levels of HK2, PKM2, GLUT1 and LDHA in HFs treated with IR780 (*n* = 3). ^*^p < 0.05, ^**^p < 0.01, ^***^p < 0.001. *Ctrl* control, *2DG* 2-deoxy-d-glucose, *TGF-β1* transforming growth factor-β1, *α-SMA* alpha-smooth muscle actin, *COL1A1* collagen type I alpha1, *GAPDH* glyceraldehyde-3-phosphate dehydrogenase, *HK2* hexokinase-II, *PKM2* pyruvate kinase isozyme M2, *GLUT1* glucose transporter-1, *LDHA* lactate dehydrogenase A

Excessive fibrosis is the most typical characteristic and the main therapeutic target of HS formation. We hypothesized that IR780 could reduce the level of fibrotic activity of fibroblasts in HS. Firstly, the biocompatibility of IR780 to fibroblasts was examined by CCK8 kits (Supplementary Fig. S4, see online supplementary material). The cytotoxicity of IR780 was not significant up to a concentration of 10 μM, but rose sharply with concentrations from 10 μM to 20 μM. Therefore, subsequent *in vitro* experiments were conducted at 10 μM. To study the fibrotic-controlling effect of IR780 on HFs and KFs, we first detected the expression of fibrosis-related factors (α-SMA, COL1A1 and fibronectin) after incubation with IR780 for 24 h. IR780 reduced the expression of these factors at the levels of mRNA and protein, thus indicating the ability of IR780 to control fibrosis (Figure 5a, b; Figure S5c). Furthermore, IR780 strongly alleviated TGF-β1-induced fibrosis in fibroblasts (Figure 5c, d; Figure S5d). As IR780 achieved a similar effect to 2DG, we hypothesized that IR780 might mitigate fibrosis by affecting glycolysis. This hypothesis was then verified by detecting the impact of IR780 on the expression of factors related to glycolysis (Figure 5e, f; Figure S5e). These results suggested that IR780 can effectively reduce the mRNA and protein levels of these factors. Thus, IR780 could be used as a novel inhibitor of glycolysis and can regulate fibrotic activity in the fibroblasts of HS by affecting glycolysis.

### Preferential accumulation of IR780 targets highly glycolytic fibroblasts

**Figure 6.**
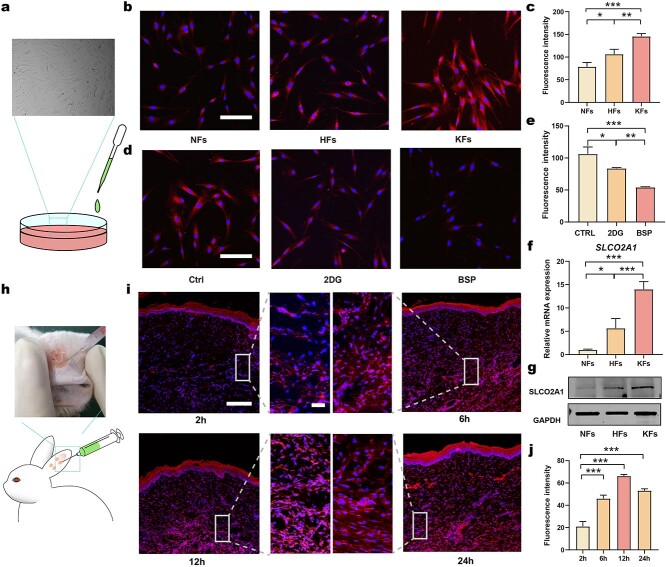
Preferential accumulation of IR780 in HFs and HS tissues. (**a**) Schematic of *in vitro* IR780 accumulation; (**b**, **c**) CLSM images and quantitation analysis of IR780 in NFs, HFs and KFs (scale bar: 150 μm); (**d**, **e**) CLSM images and quantitation analysis of IR780 uptake by HFs treated with 2DG and BSP (scale bar: 150 μm); (**f**, **g**) mRNA levels and protein levels of SLCO2A1 in NFs, HFs and KFs; (**h**) schematic of *in vivo* IR780 accumulation; (**i**, **j**) CLSM images and quantitation analysis of *in vivo* IR780 uptake 2, 6, 12 and 24 h after injection (scale bar: 200 μm, 25 μm in inset, *n* = 3). ^*^p < 0.05, ^**^p < 0.01, ^***^p < 0.001. *Ctrl* control, *2DG* 2-deoxy-d-glucose, *BSP* sulfobromophthalein disodium salt hydrate, *SLCO2A1* solute carrier organic anion transporter family member 2A1, *NFs* normal skin fibroblasts*, HFs* hypertrophic scar fibroblasts*, KFs* keloid fibroblasts, *CLSM* confocal laser scanning microscopy

Traditional glycolytic inhibitors can exert serious dose-limiting side-effects due to their non-specific nature. Herein, we investigated whether IR780 accumulates preferentially in activated fibroblasts by performing *in vitro* and *in vivo* studies (Figure 6a). First, we confirmed the preferential accumulation of IR780 in different fibroblasts, and the results showed that IR780 had a tendency to accumulate in the fibroblasts of HS and KS rather than those of NS (Figure 6b, c). Furthermore, we investigated the mechanism underlying such preferential accumulation. IR780 internalization was considered to be dependent on metabolism since the uptake was suppressed after treatment with 2DG (Figure 6d, e). Furthermore, the accumulation of IR780 was also significantly inhibited after treatment with BSP (a competitive inhibitor of OATPs), thus suggesting that the preferential uptake of IR780 was mediated by OATPs (Figure 6d, e; Figure S6, see online supplementary material). The upregulated expression of OATPs (SLCO2A1) in HFs and KFs when compared to NFs was then identified at the mRNA and protein levels (Figure 6f, g; Figure S7, see online supplementary material), thus explaining why IR780 accumulated differently in these fibroblasts. Furthermore, we also studied the *in vivo* preferential accumulation of IR780 via intralesionally injecting IR780 into rabbit HS models, and most IR780 fluorescence was aggregated in the highly hypertrophied region (Figure 6i, j). As the highly fibrotic cells manifested augmented glycolysis, these results also indicated that IR780 could specifically aggregate in highly glycolytic cells.

**Figure 7.**
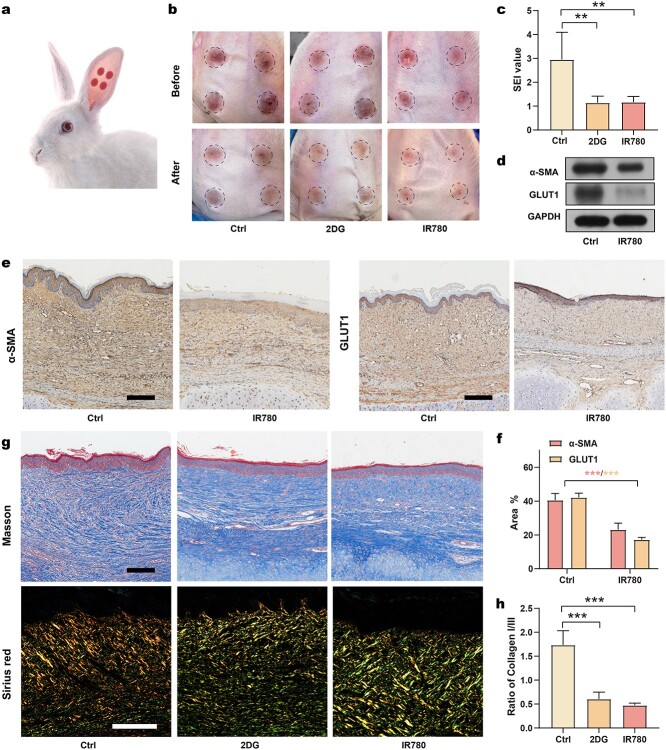
*In vivo* efficacy of IR780 in controlling HS formation. (**a**) Schematic of rabbit HS model; (**b**) appearance changes after treatment with 2DG and IR780; (**c**) SEI values of different groups (*n* = 5); (**d**) determination of protein levels of α-SMA, GLUT1 and GAPDH after IR780 treatment; (**e**) IHC analysis of α-SMA and GLUT1 in HS (scale bar: 200 μm); (**f**) quantitation analysis of expression of α-SMA and GLUT1 (*n* = 3); (**g**) Masson’s trichrome staining and Sirius red staining of HS (scale bar: 200 μm); (**h**) **s**tatistical analysis of the ratio of collagen I to collagen III of HS (*n* = 3). ^**^p < 0.01, ^***^p < 0.001. *Ctrl* control, *2DG* 2-deoxy-d-glucose, *SEI* scar elevation index, *α-SMA* alpha-smooth muscle actin, *GLUT1* glucose transporter-1, *GAPDH* glyceraldehyde-3-phosphate dehydrogenase

In conclusion, we showed that IR780 can specifically target the activated and highly glycolytic cells through OATPs-mediated transmembrane transport, and this preferential accumulation can be regulated by glycolytic metabolism.

### *In vivo* efficacy of controlling HS formation via IR780

We investigated whether IR780 can control HS formation by performing intralesional injections in a rabbit HS model (Figure 7a). After re-epithelialization (before treatment), the HS of all groups was dark red in color. After treatment, HS tissues from the IR780- and 2DG-treated groups exhibited alleviated redness and reduced thickening compared to the control group (Figure 7b). The scar elevation index (SEI), the ratio of the total HS tissue thickness to the normal skin thickness, was calculated to quantify scar thickness (Figure 7c, Supplementary Table S3, see online supplementary material). The SEI value of the R780 group and the 2DG group significantly decreased compared to the control group, thus verifying their efficiency in controlling HS formation. To investigate the mechanisms underlying these changes after IR780 treatment, we next analyzed the protein expression of GLUT1 and α-SMA by performing western blotting. A down-regulation of glycolysis and fibrosis following IR780 treatment was observed (Figure 7d). Western blotting findings were confirmed by the IHC staining of α-SMA and GLUT1, thus confirming that the glycolytic and fibrotic phenotype was significantly reduced by IR780 treatment (Figure 7e, f). To further evaluate the collagen deposition in HS tissues, we performed histological analysis using tissues stained with Masson’s trichrome and Sirius red. Masson’s staining results demonstrated abundant collagen deposition with uneven arrangement in the control group. Following treatment with 2DG or IR780, collagen deposition was remarkably decreased and the collagen fibers were remodeled parallel (Figure 7g). Besides, the ratio of collagen I:III is an important indicator reflecting the risk of HS formation: a higher ratio indicates higher risk. As a result, Sirius red staining was performed to investigate the distribution of collagen I (bright yellow) and collagen III (green) in HS tissue (Figure 7g). After treatment with IR780 and 2DG, more green fibers were observed in comparison with the control group, and the ratio of collagen I:III was significantly reduced, suggesting improved levels of collagen deposition after treatment (Figure 7h). Overall, IR780 was confirmed to control HS formation by regulating glycolysis *in vivo.*

## Discussion

HS results from excessive fibrosis of abnormally activated fibroblasts and is the most common complication after burns and trauma [1,3,4]. Recent studies showed that glycolytic dysregulation can exert an impact on the fibrotic behavior of activated fibroblasts in several fibrotic diseases and may represent a potential therapeutic target [6–8]. However, few studies have investigated the specific role of glycolysis during HS formation. In this study, we focused on the altered glycolysis during HS formation, including its augmented activity, differential expression within lesion areas and potential impact on fibrosis. In addition, this study introduced IR780 as a candidate to control HS formation. We proved that IR780 has the ability to regulate glycolysis and can specifically target activated fibroblasts showing both augmented glycolysis and fibrotic activity. Therefore, IR780 can specifically control fibrosis in activated fibroblasts and effectively control HS formation.

In this study, we demonstrated that HFs exhibit characteristics of augmented glycolysis and heterogeneity, similar to KFs. Within a lesion, only a certain proportion of fibroblasts are activated and present with high levels of glycolysis and fibrosis. Interestingly, these activated fibroblasts are arranged in clusters that are mainly localized in the superficial dermis layer in the peripheral region of scars. Notably, the glycolysis activity of HS is significantly lower than that of KS, which may be due to the different proportions of activated fibroblasts in these two types of scars. There are more activated fibroblasts among KFs in comparison with HFs. Therefore, KS has a higher tendency to expand in comparison with HS. From this perspective, monitoring glycolytic activity could be a useful indicator for the progression and prognosis of pathological scars.

We also demonstrated that augmented glycolysis is required for the excessive fibrotic phenotype in HS and that the inhibition of glycolysis can control the fibrotic behavior of activated fibroblasts. The pro-fibrotic effect of glycolysis may be because glycolysis can provide activated fibroblasts with adequate energy to support the high demand of ECM synthesis. In addition, highly glycolytic NFs may be capable of creating a metabolically favorable microenvironment, through secreting lactate and other glycolytic intermediates, to cause pathologic changes in adjacent cells. In our study, IR780 exhibited similar ability to 2DG both *in vitro* and *in vivo*. It could down-regulate glycolysis activity and thus control fibrosis and improve the characteristics of HS tissues in rabbit models. Moreover, the ability of cells to migrate and contract was also suppressed by 2DG or IR780 treatment in scratch assays, which further proved that the activity of fibroblasts was reduced via regulating glycolysis. Collectively, this evidence highlights the value of IR780 in the control of HS formation. More importantly, IR780 appears to be superior to 2DG for its preferential accumulation in activated fibroblasts, which can help to precisely regulate the augmented glycolysis without side-effects. Preferential accumulation is thought to be achieved by OATPs-mediated transmembrane transport and can be regulated by glycolysis activity. Notably, there is a synchronized up-regulation of glycolysis and OATPs in HS, which might be regulated by the highly expressed HIF-1α (Hypoxia Inducible Factor-1alfa) [19,24,25].

In this study, IR780 was applied after re-epithelialization was completed in the HS model. IR780 intervention is not recommended prior to re-epithelialization since the wound healing process requires an adequate level of glycolysis to satisfy the reparative needs of defective tissue [14]. Moreover, IR780 would not be suitable for excessive scarring. Firstly, IR780 cannot reduce ECM that has already been generated and deposited. Secondly, an abundant ECM will also prevent a drug from diffusing adequately into the lesion [26]. Therefore, the use of IR780 is more appropriate to control scarring immediately after the period of re-epithelialization.

There are several mechanisms that could underlie the ability of IR780 to regulate glycolysis and HS formation. Firstly, data suggests that IR780 is more potent than 2DG. Several key factors involved in the glycolysis pathway were all found to be suppressed by IR780 treatment. These clues indicated that IR780 may interfere with the upstream signaling pathways of glycolysis. Earlier investigations of pulmonary fibrosis found that TGF-β induced GLUT1 and glycolytic enzymes through Smad-dependent and Smad-independent pathways [27,28]. Our present research showed that IR780 diminished the induction of TGF-β-stimulated fibrotic activity in fibroblasts. Our data suggests that IR780 may regulate glycolysis by interfering with the TGF-β/SMAD signaling pathway. As previous studies showed that IR780 could selectively accumulate in the mitochondria of highly glycolytic cells, the targeting of mitochondria may also be a possible mechanism for its ability to regulate glycolysis [22].

Collectively, our results show that the excessive fibrosis of HS is associated with high levels of glycolysis and that glycolysis regulation with IR780 may represent a promising strategy for controlling fibrosis and HS formation. IR780 can target activated fibroblasts via OATPs and selectively disturb the up-regulation of glycolysis in activated fibroblasts. In this way, fibrotic activity could be suppressed and HS formation controlled.

## Conclusions

As yet, there is no optimal treatment for HS. We believe that the modulation of activated fibroblasts may hold the key to control HS formation. Our study proposes a new strategy for controlling fibrosis and HS formation based on glycolysis regulation. By targeting the activated fibroblasts and regulating their glycolysis, IR780 represents an ideal choice for controlling fibrosis and shows significant potential for controlling HS formation.

## Abbreviations

BSP: Sulfobromophthalein disodium salt hydrate; CLSM: Confocal laser scanning microscopy; COL1A1: Collagen type I alpha1; DAPI: 4′,6-Diamidino-2-phenylindole; 2DG: 2-Deoxy-d-glucose; DMEM: Dulbecco’s modified Eagle’s medium; GAPDH: Glyceraldehyde-3-phosphate dehydrogenase; GLUT1: Glucose transporter-1; HFs: Hypertrophic scar fibroblasts; HIF-1alfa: Hypoxia Inducible Factor-1alfa; HK2: Hexokinase-II; HS: Hypertrophic scar; IF: Immunofluorescence; IHC: Immunohistochemistry; KFs: Keloid fibroblasts; KS: Keloid scar; LDHA: Lactate dehydrogenase A; NFs: Normal skin fibroblasts; NIR: Near-infrared; NS: Normal skin; OATPs: Organic anion transporter peptides; PKM2: Pyruvate kinase isozyme M2; SEI: Scar elevation index; siRNA: Small interfering RNA; SLCO2A1: Solute carrier organic anion transporter family member 2A1; α-SMA: alpha-Smooth muscle actin; TGF-β1: Transforming growth factor-β1.

## Availability of data and materials

The data used in this publication is available upon request.

## Ethics approval and consent to participate

All patients sign informed consent and all procedures were approved by the Ethics Committee of Shanghai Ninth People's Hospital, School of Medicine, Shanghai Jiao Tong University, Shanghai, China (SH9H-2022-TK332-1). All animal experiments were approved by Ethics Committee of Shanghai Ninth People's Hospital, School of Medicine, Shanghai Jiao Tong University, Shanghai, China and were handled according to international animal welfare standards (SH9H-2022-A612-SB).

## Authors’ contributions

YSC and ZZ contributed to the study concept and design. XXM and ZXY performed the experiments and assembled the data. WYX and JC performed the statistical analysis. SF and PRM did the investigation. XXM drafted the manuscript. YSC, ZZ and YXZ supervised the writing of the manuscript and critically revised it for important intellectual content. All authors read and approved the final manuscript.

## Supplementary Material

Figure_S1_tkac015

Figure_S2_tkac015

Figure_S3_tkac015

Figure_S4_tkac015

Figure_S5_tkac015

supporting_information_tkac015
