# Supplementary figures and images for: Control of fibrosis and hypertrophic scar formation via glycolysis regulation with IR780
Source: Burns Trauma. 2022 Jun 24;10:tkac015. doi: 10.1093/burnst/tkac015 (PMC9227726; doi:10.1093/burnst/tkac015)

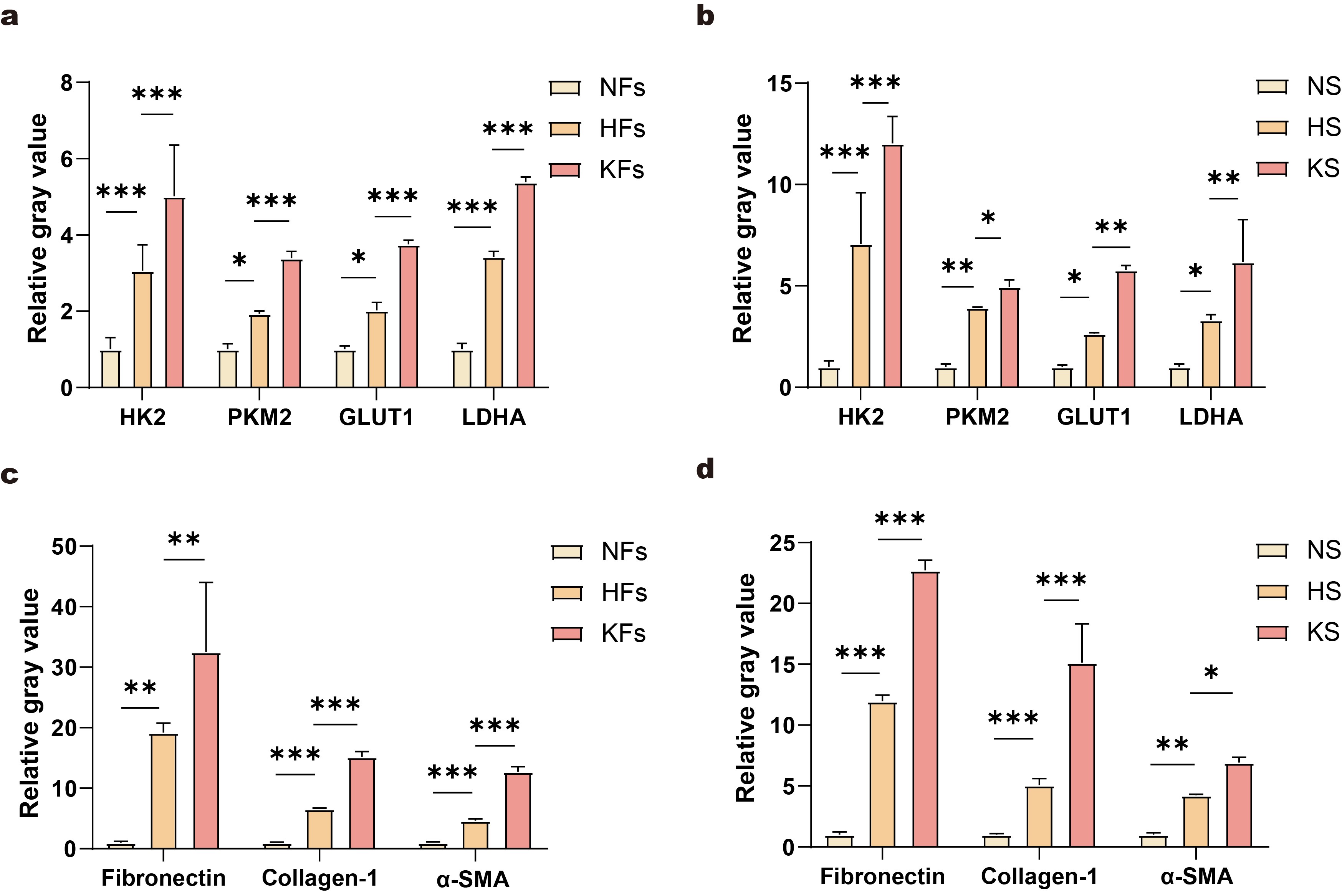

Supplement: Figure_S1_tkac015 [file figure_s1_tkac015.jpeg]

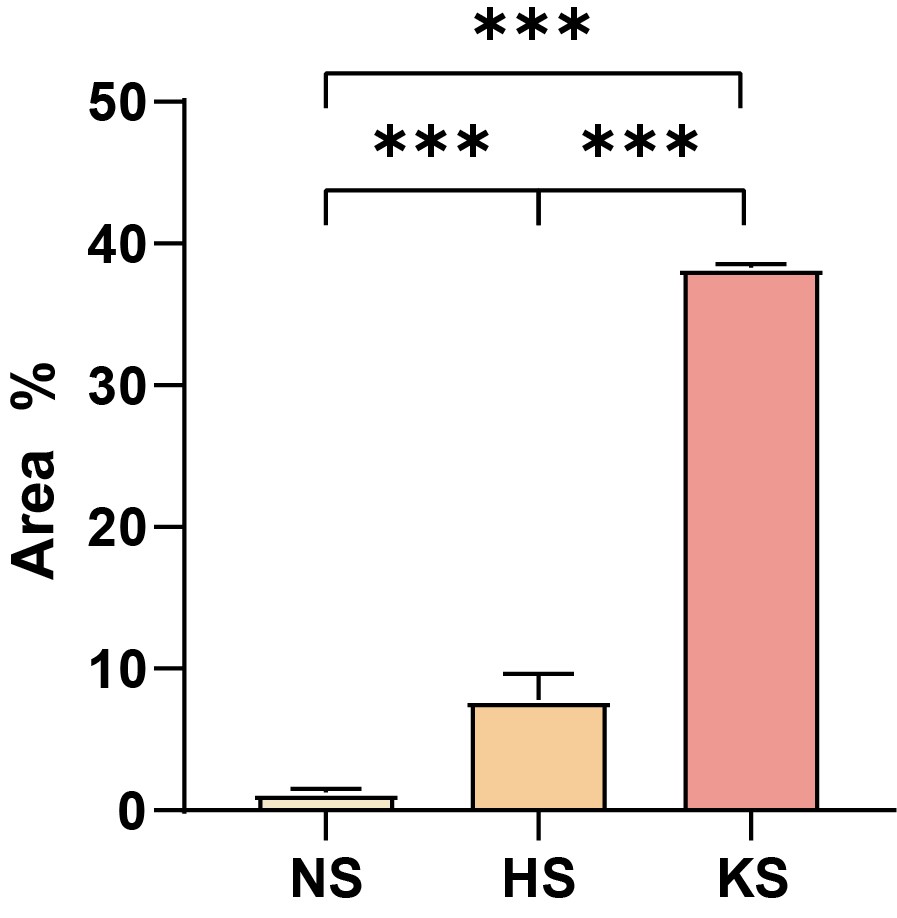

Supplement: Figure_S2_tkac015 [file figure_s2_tkac015.jpeg]

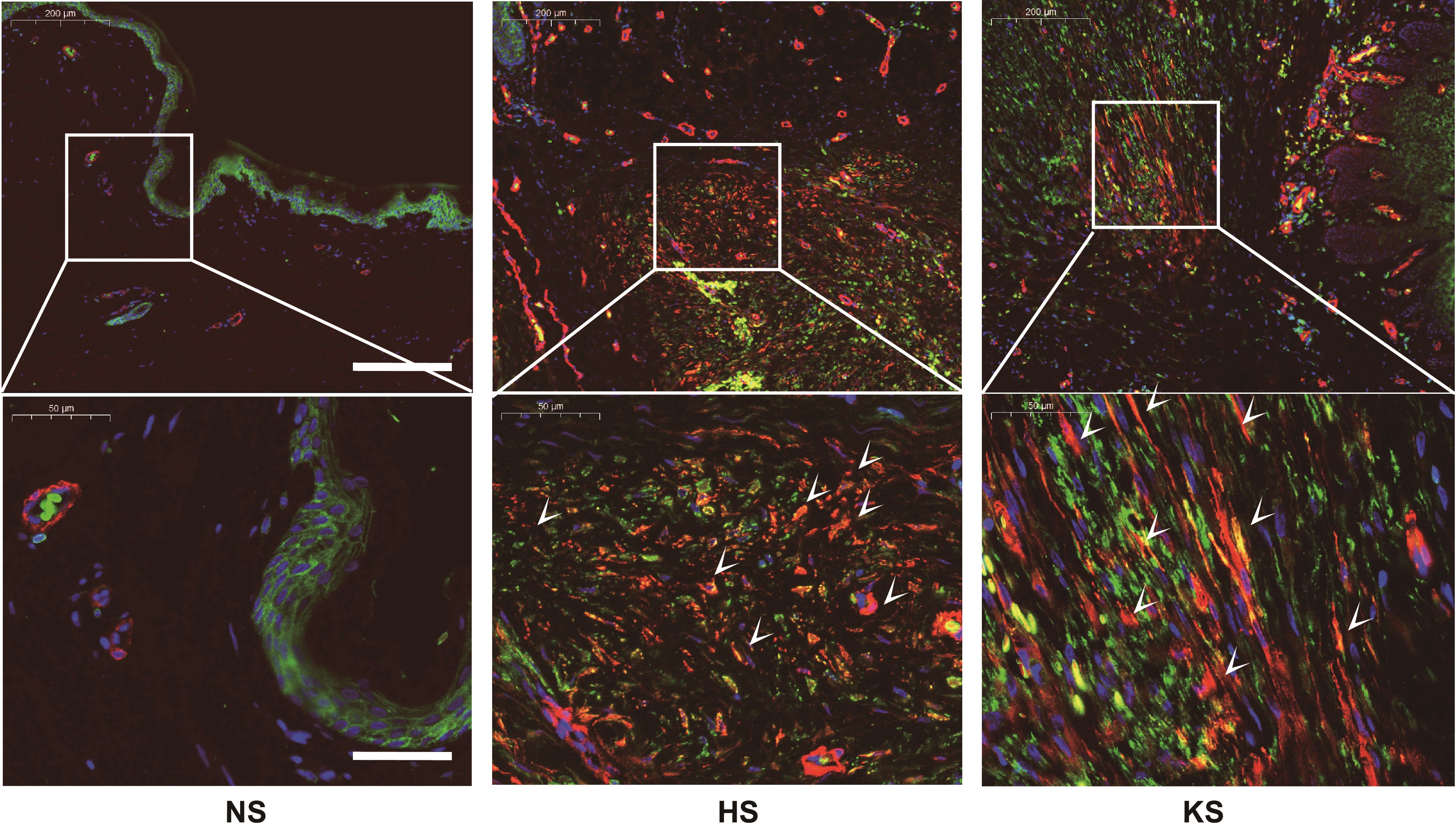

Supplement: Figure_S3_tkac015 [file figure_s3_tkac015.jpeg]

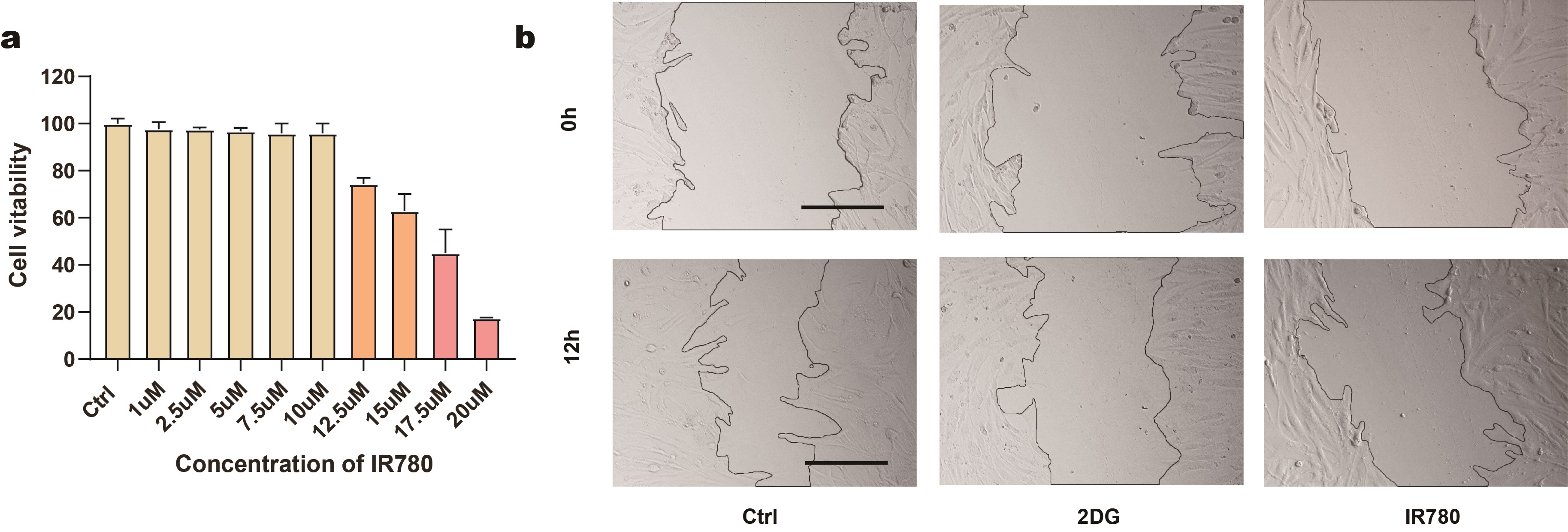

Supplement: Figure_S4_tkac015 [file figure_s4_tkac015.jpeg]

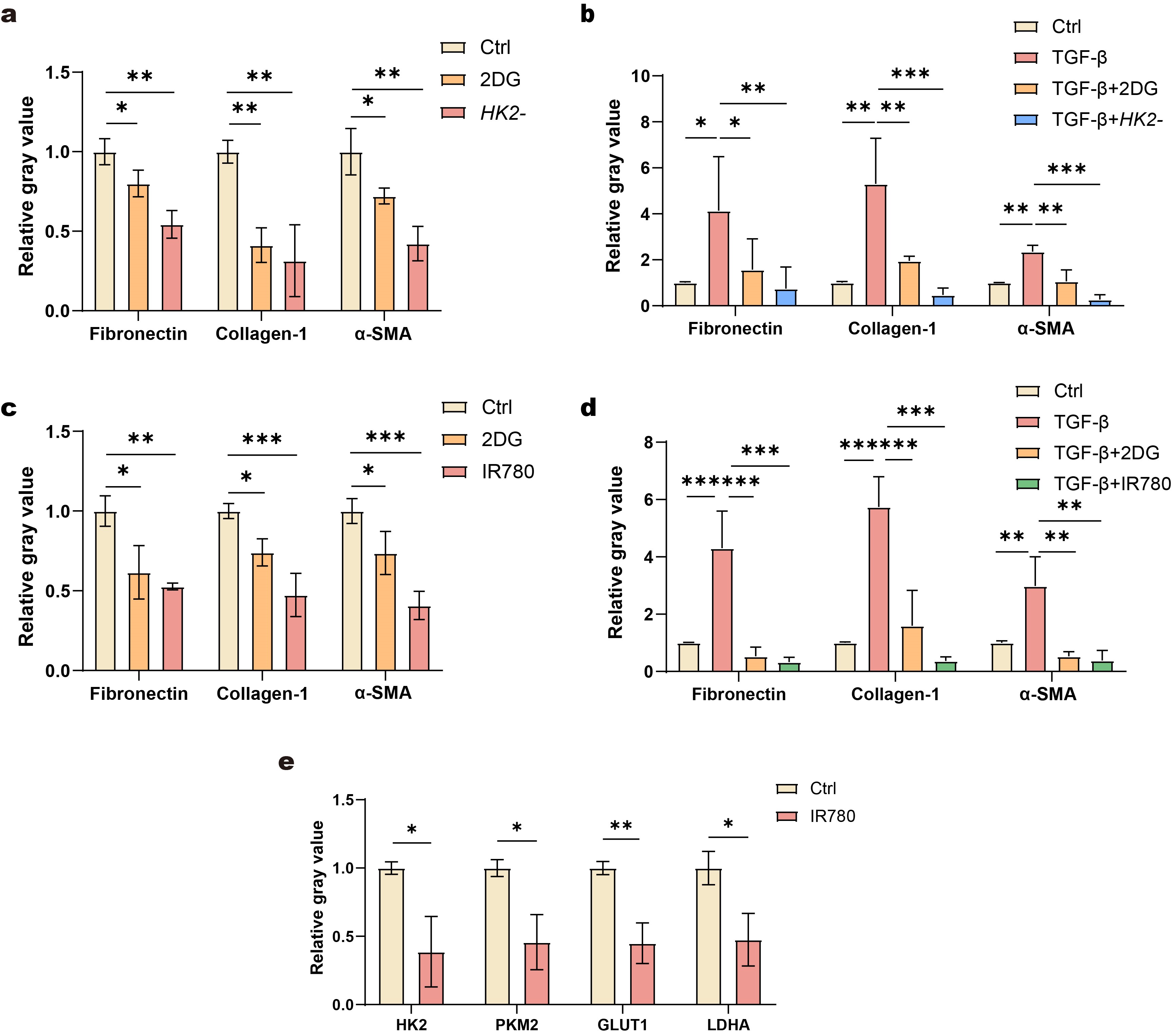

Supplement: Figure_S5_tkac015 [file figure_s5_tkac015.jpeg]
