# Supplementary material for: Control of fibrosis and hypertrophic scar formation via glycolysis regulation with IR780
Source: Burns Trauma. 2022 Jun 24;10:tkac015. doi: 10.1093/burnst/tkac015 (PMC9227726; doi:10.1093/burnst/tkac015)
Supplement: supporting_information_tkac015 [file supporting_information_tkac015.docx]

Supporting Information for

**Control of Fibrosis and Hypertrophic Scar Formation *via* Glycolysis Regulation with IR780**

**Table S1**. The sequence for primers for quantitative real-time PCR

| **Gene** |  | **Primer sequence** |
| --- | --- | --- |
| *β-Actin* | Forward | 5’ AAGGTGACAGCAGTCGGTT 3’ |
|  | Reverse | 5’ TGTGTGGACTTGGGAGAGG 3’ |
| *HK2* | Forward | 5’ ACTGGACTGGGTTTTGTCTC 3’ |
|  | Reverse | 5’ GGCAAGGGGGATTACTAAG 3’ |
| *PKM2* | Forward | 5’ GATTCACCACCCATCACAG 3’ |
|  | Reverse | 5’ GCCACATTCATTCCAGACTTA 3’ |
| *LDHA* | Forward | 5’ TAGCAGATTTGGCAGAGAGTAT 3’ |
|  | Reverse | 5’ CAAGGAACACTAAGGAAGACA 3’ |
| *GLUT1* | Forward | 5’ AGTATGTGGAGCAACTGTGTG 3’ |
|  | Reverse | 5’ TGAAGTAGGTGAAGATGAAGAAC 3’ |
| *COL1A1* | Forward | 5’ CAGCCGCTTCACCTACAGC 3’ |
|  | Reverse | 5’ TTTTGTATTCAATCACTGTCTTGCC 3’ |
| *Fibronectin* | Forward | 5’ GGAGAATTCAAGTGTGACCCT 3’ |
|  | Reverse | 5’ TGCCACTGTTCTCCTACGTGG 3’ |
| *α-SMA* | Forward | 5’ AAAGCAAGTCCTCCAGCGTT 3’ |
|  | Reverse | 5’ GCTTCACAGGATTCCCGTCT 3’ |
| *SLCO2A1* | Forward | 5’ AGAACCCCCAGAAGGAGAC 3’ |
|  | Reverse | 5’ AAATGGCTGAATAGGCACTGT 3’ |

*HK2* hexokinase-II, *PKM2* pyruvate kinase isozyme M2, *LDHA* lactate dehydrogenase A, *GLUT1* glucose transporter-1, *COL-A1* collagen type I alpha1, *α-SMA* alpha smooth muscle actin, *SLCO2A1* Solute Carrier Organic Anion Transporter Family Member 2A1

**Table S2.** The sequence for siRNA

| **Sequences of siRNA** | | |
| --- | --- | --- |
| *HK2* | SS sequence | CACGAUGAAAUUGAACCUGGU |
|  | AS sequence | ACCAGGUUCAAUUUCAUCGUG |

*HK2* hexokinase-II, *SS* sense strand, *AS* antisense strand

**Table S3**. SEI value of different groups

| **Ctrl** | **2DG** | **IR780** |
| --- | --- | --- |
| 3.152874452 | 1.045062696 | 1.080307637 |
| 4.864522417 | 0.978332908 | 0.935157895 |
| 2.123540258 | 1.632536159 | 1.472908864 |
| 2.188649501 | 0.98768316 | 1.367213115 |
| 2.410937801 | 1.062551896 | 0.97235023 |

*Ctrl* control, *2DG* 2-deoxy-d-glucose


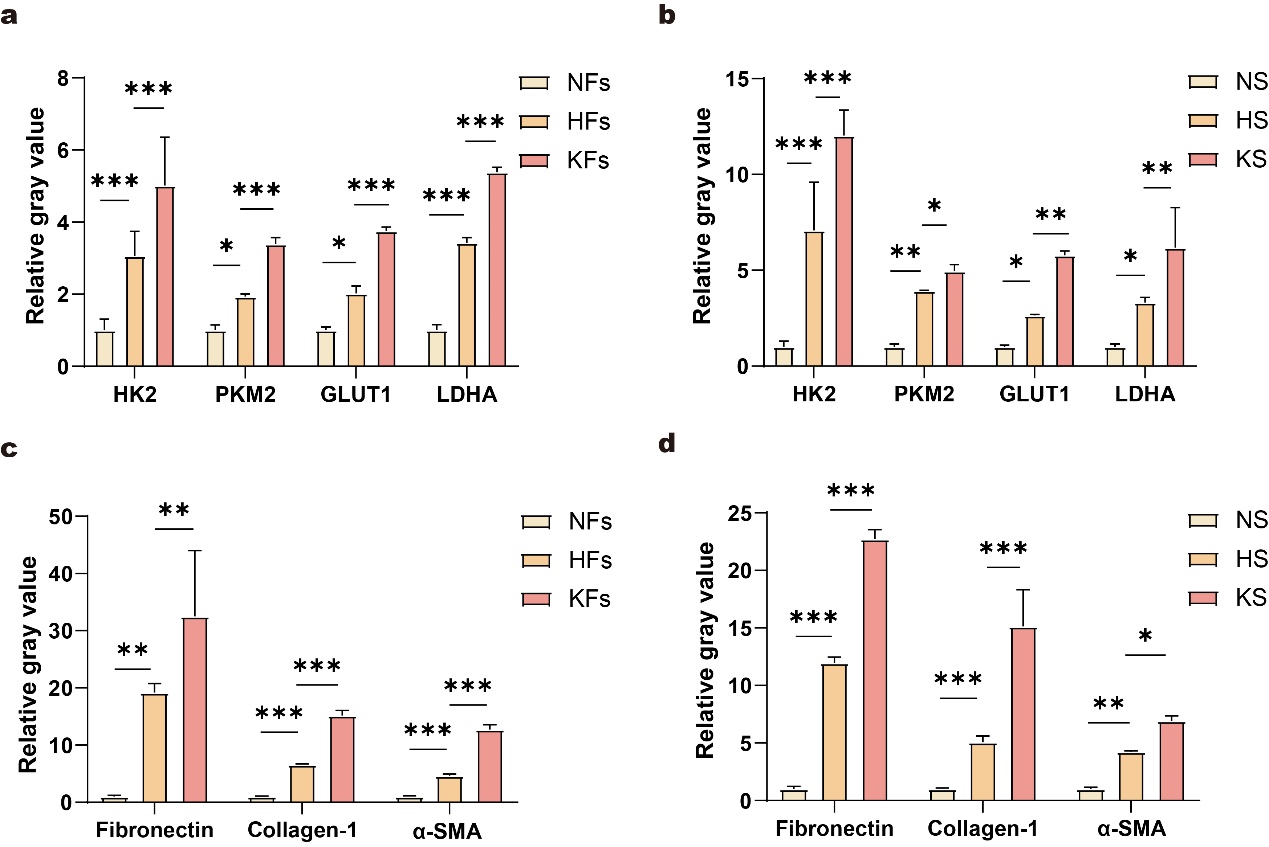


**Figure S1.** Quantitation analysis of protein levels of HK2, PKM2, GLUT1 and LDHA in (**a**) NFs, HFs, KFs and (**b**) in tissue samples of NS, HS, and KS; Quantitation analysis of protein levels of α-SMA, Collagen-1, Fibronectin in (**c**) NFs, HFs, KFs and (**d**) in tissue samples of NS, HS, and KS. (n = 3).  ^*^*p*< 0.05, ^**^*p*< 0.01,^***^*p* < 0.001. *HK2* hexokinase-II, *PKM2* pyruvate kinase isozyme M2, *GLUT1* glucose transporter-1, *LDHA* lactate dehydrogenase A, *α-SMA* alpha smooth muscle actin, *NFs* normal skin fibroblasts*, HFs* hypertrophic scar fibroblasts*, KFs* keloid fibroblasts*, NS* normal skin*, HS* hypertrophic scar*, KS* keloid scar


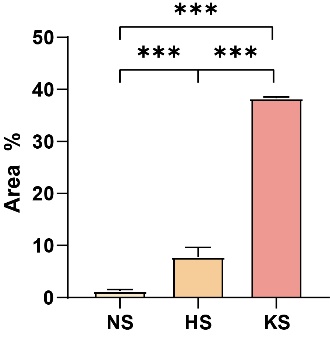


**Figure S2**. Quantitation analysis of IF staining of GLUT1 in tissue samples of NS, HS, and KS. (n = 3). ^***^*p* < 0.001. *GLUT1* glucose transporter-1, *NS* normal skin*, HS* hypertrophic scar*, KS* keloid scar


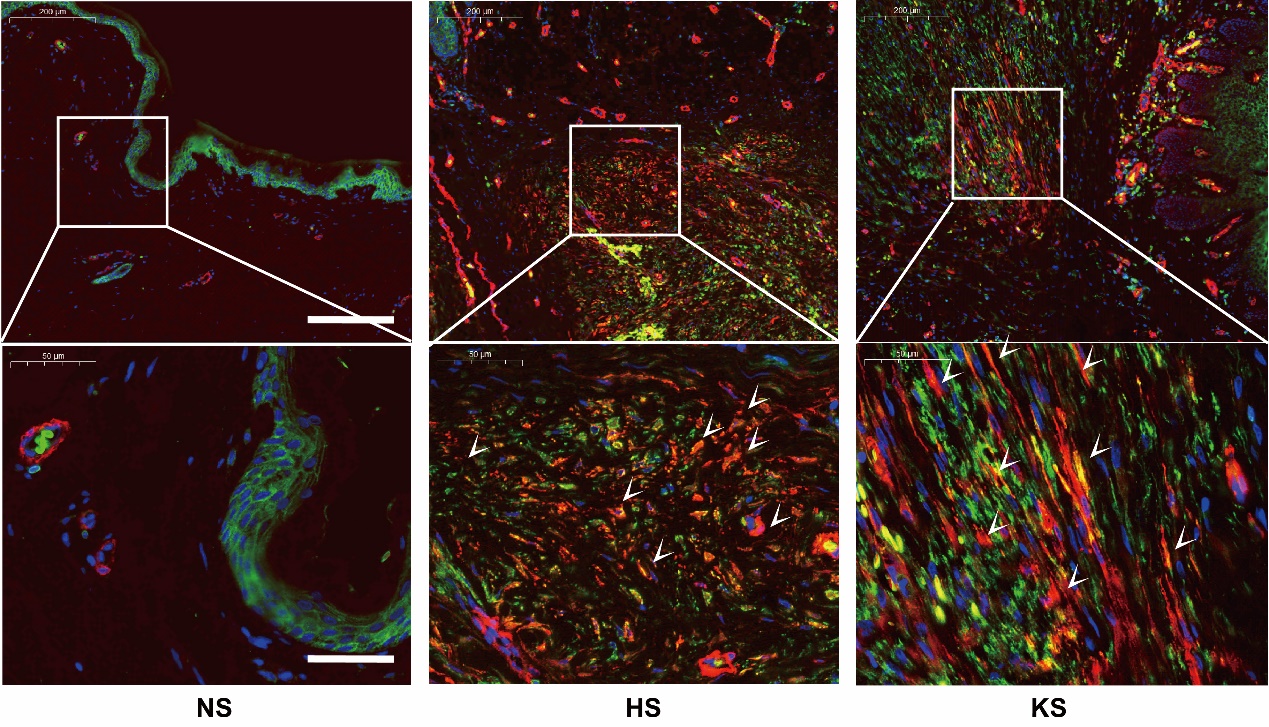


**Figure S3.** IF colocalization of GLUT-1 (green) and α-SMA (red) in NS, HS and KS. The colocalization was shown in orange and are pointed by white arrows. (Scale bar: 200 μm, 50 μm )。 *GLUT1* glucose transporter-1, *α-SMA* alpha smooth muscle actin, *NS* normal skin*, HS* hypertrophic scar*, KS* keloid scar


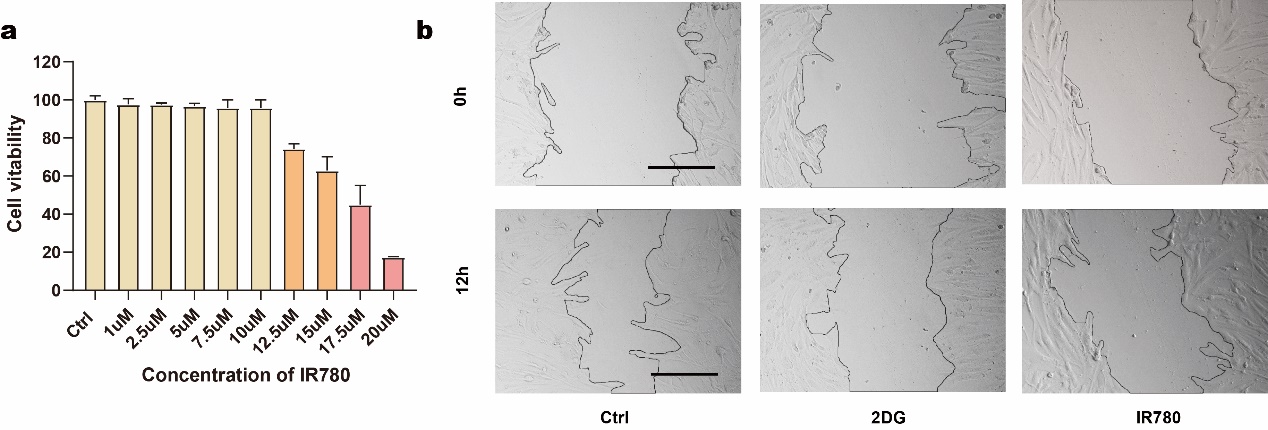


**Figure S4**. Biocompatibility of IR780 **(a)** CCK-8 assay indicating there was no significant difference in cell proliferation with the concentration under 10uM ( *p* > 0.05, n = 3). **(b)** Typical photographs of cell migration showing more fibroblasts migrate to the scratch in the control group than the 2DG and IR780 treated group (Scale bar: 200 μm). *CCK-8* cell counting kit 8, *Ctrl* control, *2DG* 2-deoxy-d-glucose


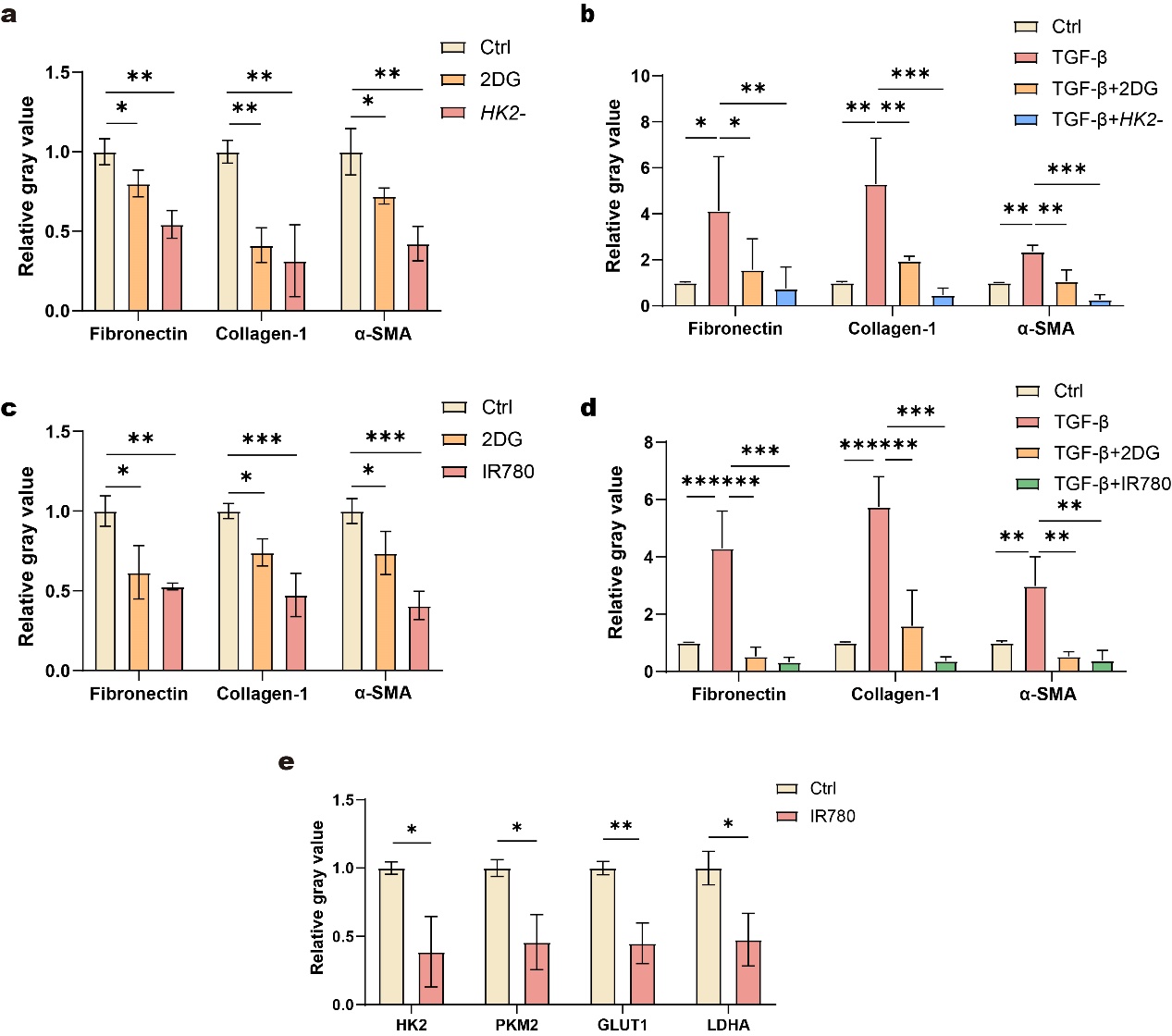


**Figure S5**. Variation of protein levels of α-SMA, Collagen-I and Fibronectin after treatment with 2DG and siRNA targeting *HK2* in (**a**) HFs and (**b**) HFs activated with TGF-β1; Variation of protein levels of protein levels of α-SMA, Collagen-1 and Fibronectin after treatment with 2DG and IR780 in (**c**) HFs and (**d**) HFs activated with TGF-β1; **(e)** Quantitation analysis of protein levels of HK2, PKM2, GLUT1 and LDHA in HFs treated with IR780 (n = 3).  ^*^*p*< 0.05, ^**^*p*< 0.01,^***^*p* < 0.001. *HK2* hexokinase-II, *PKM2* pyruvate kinase isozyme M2, *GLUT1* glucose transporter-1, *LDHA* lactate dehydrogenase A, *α-SMA* alpha smooth muscle actin, *2DG* 2-deoxy-d-glucose, *TGF-β1* transforming growth factor-β1, *HK2-* Small interfering RNA targeting hexokinase-II, *NFs* normal skin fibroblasts*, HFs* hypertrophic scar fibroblasts*, KFs* keloid fibroblasts


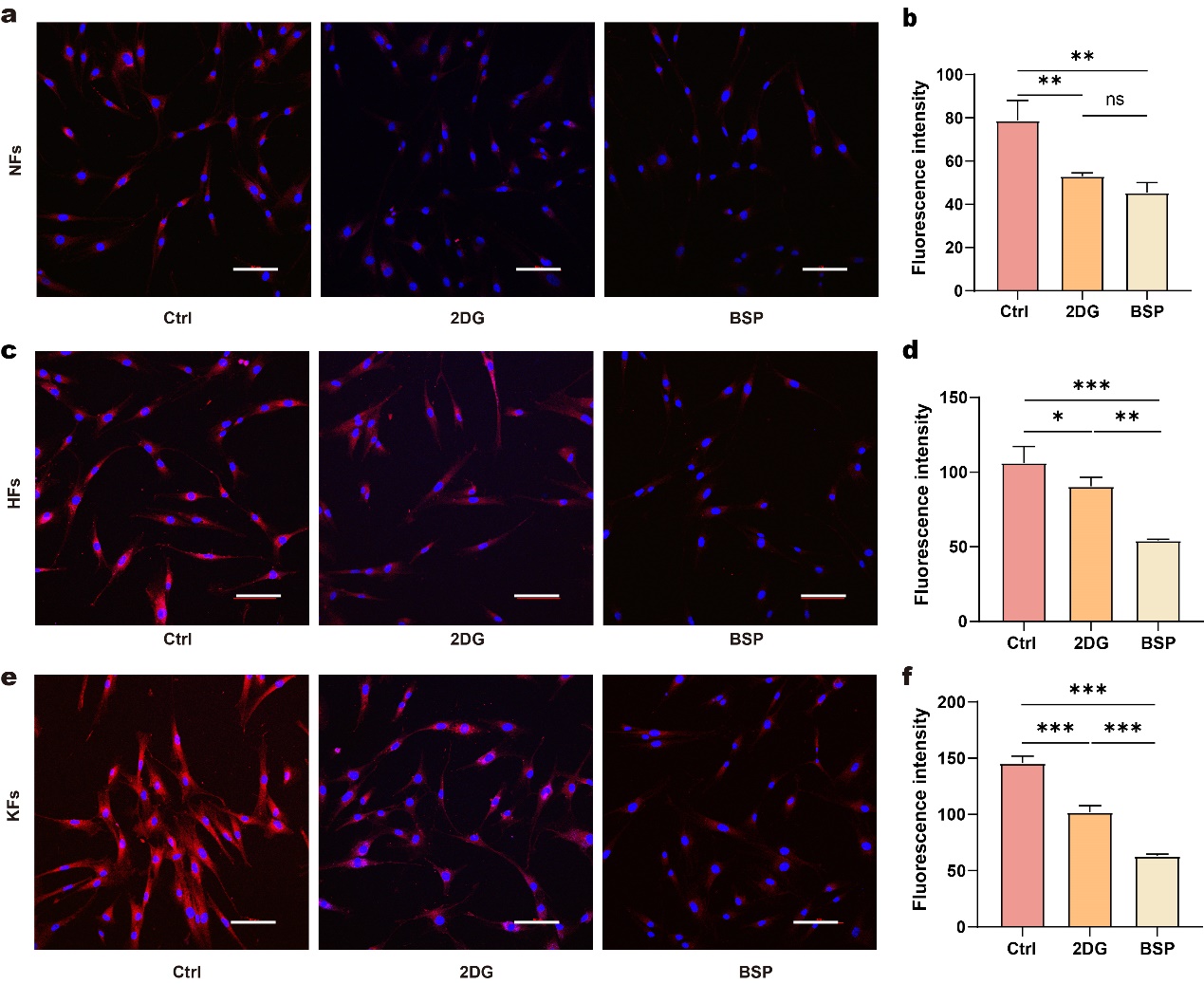


**Figure S6**. CLSM images and quantitation analysis of IR780 uptake after treatment with 2DG and BSP in **(a and b)** NFs, **(c and d)** HFs and **(e and f)** KFs. **(**Scale bar: 100 μm, n = 3) *CLSM* confocal laser scanning microscopy**,** *Ctrl* control, *2DG* 2-deoxy-d-glucose, *BSP* sulfobromophthalein disodium salt hydrate, *NFs* normal skin fibroblasts*, HFs* hypertrophic scar fibroblasts*, KFs* keloid fibroblasts


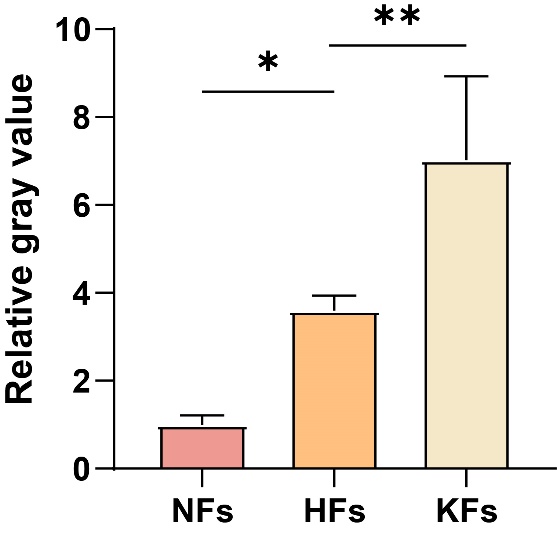


**Figure S7**. Quantitation analysis of protein levels of SLCO2A1 in NFs, HFs and KFs. (n = 3) *SLCO2A1* Solute Carrier Organic Anion Transporter Family Member 2A1, *NFs* normal skin fibroblasts*, HFs* hypertrophic scar fibroblasts*, KFs* keloid fibroblasts
